# Development of a Modular Vaccine Platform for Multimeric Antigen Display Using an Orthobunyavirus Model

**DOI:** 10.3390/vaccines9060651

**Published:** 2021-06-15

**Authors:** Andrea Aebischer, Kerstin Wernike, Patricia König, Kati Franzke, Paul J. Wichgers Schreur, Jeroen Kortekaas, Marika Vitikainen, Marilyn Wiebe, Markku Saloheimo, Ronen Tchelet, Jean-Christophe Audonnet, Martin Beer

**Affiliations:** 1 Friedrich-Loeffler-Institut, 17493 Greifswald-Insel Riems, Germany; andrea.aebischer@fli.de (A.A.); kerstin.wernike@fli.de (K.W.); patricia.koenig@fli.de (P.K.); kati.franzke@fli.de (K.F.); 2 Laboratory of Virology, Wageningen Bioveterinary Research, 8221 RA Lelystad, The Netherlands; paul.wichgersschreur@wur.nl (P.J.W.S.); jeroen.kortekaas@wur.nl (J.K.); 3 VTT Technical Research Centre of Finland Ltd., 02150 Espoo, Finland; Marika.Vitikainen@vtt.fi (M.V.); Marilyn.Wiebe@vtt.fi (M.W.); markku.saloheimo@vtt.fi (M.S.); 4 Dyadic Netherland B.V., 6709 PA Wageningen, The Netherlands; rtchelet@dyadic.com; 5 Boehringer Ingelheim Animal Health, 69800 Saint-Priest, France; Jean-Christophe.AUDONNET@boehringer-ingelheim.com

**Keywords:** emerging infectious disease, zoonosis, modular vaccine, epitope, lumazine synthase, Schmallenberg virus, SpyCatcher/SpyTag, C1 production host

## Abstract

Emerging infectious diseases represent an increasing threat to human and animal health. Therefore, safe and effective vaccines that could be available within a short time frame after an outbreak are required for adequate prevention and control. Here, we developed a robust and versatile self-assembling multimeric protein scaffold particle (MPSP) vaccine platform using lumazine synthase (LS) from *Aquifex aeolicus.* This scaffold allowed the presentation of peptide epitopes by genetic fusion as well as the presentation of large antigens by bacterial superglue-based conjugation to the pre-assembled particle. Using the orthobunyavirus model Schmallenberg virus (SBV) we designed MPSPs presenting major immunogens of SBV and assessed their efficacy in a mouse model as well as in cattle, a target species of SBV. All prototype vaccines conferred protection from viral challenge infection and the multivalent presentation of the selected antigens on the MPSP markedly improved their immunogenicity compared to the monomeric subunits. Even a single shot vaccination protected about 80% of mice from an otherwise lethal dose of SBV. Most importantly, the MPSPs induced a virtually sterile immunity in cattle. Altogether, LS represents a promising platform for modular and rapid vaccine design.

## 1. Introduction

Emerging infectious diseases represent an increasing threat for human and animal health as a consequence of economic development, increasing global commerce, travel and the ongoing disruption of ecologies. The majority of newly evolving pathogens are zoonotic viruses originating from a wildlife reservoir bringing with them the potential for pandemic spread [1,2]. The emergence of Middle East Respiratory Syndrome Coronavirus (MERS-CoV) in Saudi Arabia in 2012 [3], the re-emergence of Zika virus in the Americas [4] and most dramatically, the occurrence of SARS-CoV-2 in December 2019 [5,6,7] affirm the reality of these threats. The latter further demonstrates that despite sophisticated diagnostic and detection tools, such events are poorly predictable. Thus, the overall level of preparedness has to be further improved, and novel strategies allowing a fast reaction to combat such newly emerging pathogens are urgently needed [8,9].

Vaccination represents the most successful strategy to confer protection from infection or clinical disease and to prevent further pathogen spreading. Over the last few years, efforts enabling an accelerated production of efficient vaccines were dominated by the development of innovative delivery platforms such as recombinant virus-like particles (VLPs) [10,11]. Due to their small size and their multivalent, highly repetitive presentation of antigens, VLPs can induce a very efficient immune response in combination with an outstanding safety profile since they lack viral genomes and are unable to cause any disease [12,13,14]. In addition, they are suitable for low-cost and large-scale production without the need for biosafety facilities, which can significantly accelerate manufacturing and regulatory processes.

In the present study, we developed a modular vaccine platform for multivalent antigen display using lumazine synthase (LS) from the hyperthermophilic bacterium *Aquifex aeolicus* as a scaffold-protein (also designated as “multimeric protein scaffold particle” or “MPSP”). LS forms T = 1 icosahedrons with 60 subunits (PDB ID 1HQK; [15]) and assemblies have previously been used for multimerization of antigens and as a delivery platform for dendritic cell-based vaccines or for targeted drug delivery [16,17,18]. In recent years, a variety of strategies have been developed and applied to present antigens on VLPs, all of them with inherent advantages and disadvantages (reviewed in [19]). In our study, we directly compared two of these methods with regards to efficiency and potency. In the first approach, we genetically fused a peptide epitope in the self-assembling LS MPSP. In comparison, we employed a plug-and-display strategy and conjugated heterogeneously produced antigens to the pre-assembled scaffold by spontaneous isopeptide formation between the SpyCatcher (SpyC) protein and its peptide partner SpyTag (SpyT) [20,21]. This bioconjugation method has previously been used to design diverse particulate vaccines and to enhance their efficacy [22,23,24,25,26,27]. Since its development in 2012, the resilience, reaction speed and modular reactivity of this so-called protein superglue have been improved continuously [28,29]. The latest 003 generation allows for the binding of trimeric and even tetrameric antigens to VLPs with an exceptionally high reaction rate [30]. This novel SpyCatcher003-mi platform was also very recently used to develop a potent and promising COVID-19 vaccine candidate which induced a strong neutralizing antibody response in mice and pigs [31].

We selected Schmallenberg virus (SBV) as a model for a newly evolving and fast spreading pathogen to evaluate the functionality and applicability of our novel vaccine platform. SBV is an orthobunyavirus belonging to the family *Peribunyaviridae*. It was first detected in 2011 [32], and has subsequently caused a large epizootic in European livestock. SBV is transmitted by midges (*Culicoides* spp.) and predominately infects ruminants. While it causes only mild and unspecific disease in adult animals, infection during a critical stage of pregnancy can induce abortions and severe congenital malformations in the fetus [33,34]. 

**Figure 1:**
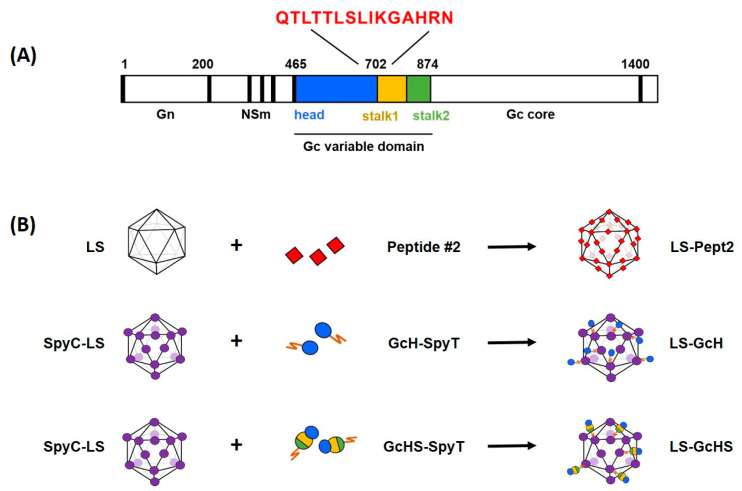
Design and generation of vaccine candidates. (**A**) Schematic presentation of the SBV M-segment showing the selected model antigens within the variable N-terminal region of the Gc ectodomain: Gc head (GcH, aa 465–702), Gc head-stalk (GcHS, aa 465–874) and peptide epitope QTLTTLSLIKGAHRN (Pept2, aa 694–708); (**B**) Illustration of the applied vaccine design strategies and the resulting candidate vaccines. Peptide2 was genetically fused into the LS MPSP and the GcH or GcHS domain equipped with a SpyT were conjugated to the SpyC-LS MPSP by spontaneous isopeptide bonding.

We previously identified the *N*-terminal region of the SBV Gc envelope protein as a potent antigen [35,36,37]. Based on the molecular architecture of this spike protein, we subsequently confirmed that the head domain (aa 465–702) as well as the complete head-stalk domain (aa 465–874) contain the major targets of neutralizing antibodies and can provide protection from SBV infection [38]. We therefore selected these two model antigens to evaluate the SpyC/SpyT-mediated plug-and-display on LS-MPSPs. For the heterogeneous expression of Gc head (GcH) and head-stalk (GcHS) domains we used the insect cell host *Drosophila S2* [39] and the thermophilic fungus C1 (*Thermothelomyces heterothallica*, formerly named *Myceliophthora thermophila*) [40], respectively. To test the genetic fusion approach in parallel, we selected a linear peptide epitope located at the interface of the SBV Gc head and stalk domain (aa 694–708) (Figure 1). Using these antigens, we designed LS MPSP-based candidate vaccines and comparatively tested their immunogenicity in vaccination-challenge studies; both in a small animal model and in cattle, one of the target species of SBV.

We demonstrate that the immunogenicity and vaccine efficacy of our selected antigens are markedly improved by a multivalent display on the newly developed LS MPSP. Thus, it represents a promising and versatile platform for the fast and rational design of modular vaccines targeting emerging infectious diseases.

## 2. Materials and Methods

### 2.1. Ethics Statement

All animal experiments were performed in accordance with the relevant regulations and were reviewed by the competent authority (State Office of Agriculture, Food Safety and Fisheries of the Mecklenburg-Vorpommern, Rostock, Germany, permission number LALLF M-VTSD/7221.3-1-067/17).

### 2.2. Cells and Viruses

BHK21 cells (CCLV 0164) and Vero 76 cells (CCLV 0228) were obtained from the Collection of Cell Lines in Veterinary Medicine, BioBank, Friedrich-Loeffler-Institut, Greifswald, Insel Riems, Germany. *Drosophila S2* cells (R69007) were purchased from Thermo Scientific and BL21 (DE3) Competent *E. coli* (C25276H) cells from New England BioLabs. C1 cells (parent strain DNL131 and production strain DNL139) are a trademark of Dyadic Netherland B.V. and have been developed and established at the VTT Technical Research Centre of Finland Ltd., Espoo, Finland.

SBV isolate BH80/11-4 (used for virus neutralization tests) was grown on BHK21 cells and SBV isolate BH619/12 (the challenge virus for mouse trials) was grown on Vero76 cells. The viruses were harvested 72h post infection by one cycle of freezing and thawing and subsequent clarification by centrifugation. Aliquots were stored at −80 °C until further use. An SBV field strain that was passaged only in cattle [41] was used for challenge infection in the cattle immunogenicity trial.

### 2.3. PepScan Analysis

PepScan analyses were performed by Pepscan Presto BV (Zuidersluisweg 2, 8243RC Lelystad, The Netherlands). Serum samples from two experimentally infected cows [37] as well as from two sheep obtained on day 0 and 28 post SBV infection were submitted for analysis. Sheep sera were kindly provided by Wageningen Bioveterinary Research, Lelystad, The Netherlands.

### 2.4. Cloning

#### 2.4.1. Construction of the SpyCatcher-Lumazine Synthase Expression Plasmid

A codon-optimized sequence (*E-coli*) of the *Aquifex aeolicus* Lumazine Synthase (NC_000918.1) was subcloned in the pET15b-expression vector (Novagen, Merck KGaA, Darmstadt, Germany). ΔN1SpyCatcher [42] was amplified from a codon-optimized synthetic gene (ThermoFisher Scientifc, Dreieich, Germany) and N-terminally fused to the LS sequence yielding SpyC-LS. An N-terminal FLAG-tag was additionally inserted for the purification of expression products. For construction of LS-particles displaying peptide epitopes, the SpyCatcher-sequence was replaced by the respective codon-optimized peptide sequence and a 9 aa-linker sequence (GSGGSGGSG) using a restriction-free cloning method [43]. All constructs were verified by Sanger sequencing.

#### 2.4.2. Constructs for Expression in Drosophila *S2* Cells

Several SpyTag-conjugated proteins were expressed in *S2* cells: SBV Gc head (aa 465–702), SBV Gc head-stalk (aa 465–874), SBV Gc core (aa 890–1330), SBV Gc stalk 1 (aa 703–798) and Akabane virus Gc head (AKAV-GcH, aa 465–701). All sequences were amplified from codon-optimized synthetic genes (ThermoFisher Scientifc) based on GenBank entries CCF55030 (SBV) and BAV17033.1 (AKAV), respectively, and cloned in the pMT *Drosophila S2* expression vector (ThermoFisher Scientifc) in frame with an N-terminal BiP secretion signal. C-terminally, a 25 aa-linker sequence (GGSQSDSRGGNGNGGGAGGNGGGSA), the SpyTag (AHIVMVDAYKPTK), a TEV cleavage site (LEENLYFQSA) and a double Strep-Tag (WSHPQFEKGGSGGGSGGSAWSHPQFEK) were added. All constructs were verified by Sanger sequencing. Primer sequences are available upon request. The 25 aa-linker was inserted between antigen and SpyTag in order to minimize sterical hinderance during the conjugation process due to the size of the antigen.

#### 2.4.3. SBV Gc Head-Stalk Construct for Expression in C1 Cells

A codon-optimized gene fragment encoding the C1 CBH1 signal sequence, the SBV Gc head-stalk domain (aa 465–874), the linker GGSQSDSRGGNGNGGGAGGNGGGSA, the SpyTag (AHIVMVDAYKPTK), a TEV cleavage site (LEENLYFQSA) and a C-tag (EPEA) were synthesized by GenScript. The fragment was subsequently amplified from the GenScript vector by PCR using primers that included 5′ and 3′ overhangs of 30 bp for cloning sites on the C1 expression vectors pMYT0133 and pMYT0134. Expression plasmids were generated using Gibson assembly cloning with NEBbuilder™ HiFi DNA Assembly (New England Biolabs, Frankfurt am Main, Germany) according to the manufacturer’s instructions. The final expression construct integrated into the C1 genome consisted of two expression cassettes in opposite orientations and the nia1-hygromycin double marker between the cassettes. The hygromycin resistance marker is split between the two expression vectors and becomes complete and functional by recombination upon transformation to C1 [44]. Both expression vectors have the AnSES synthetic promoter [45] and either the bgl8 (pMYT0133) or chi1 (pMYT0134) terminator. The final expression vectors encoding the Gc head-stalk domain were pMYT0451 (from pMYT0133) and pMYT0452 (from pMYT0134). 

### 2.5. Expression and Purification of LS Fused to SpyCatcher or to Peptides 

pET15b-expression plasmids were transformed in BL21 (DE3) competent *E. coli* (#C25276H, New England Biolabs, Frankfurt am Main, Germany). Expression was performed in 0.5 L cultures (LB Lennox medium, 100 µg/mL ampicillin). The cultures were incubated for about 2 h at +37 °C with shaking. At OD600 = 1.0 the cultures were induced with 0.5 mM IPTG and incubated for another 4 h at +30 °C with shaking. The cells were harvested by centrifugation and the pellets were frozen at −20 °C overnight and subsequently resuspended in lysis buffer consisting of 50 mM Tris-HCl, 150 mM NaCl, 1% Triton-X100 (*v*/*v*); pH 7.4, supplemented with 1 mg/mL lysozyme, 1X Protease inhibitor (#04693116001, Roche, Mannheim, Germany) and Benzonase (E1014, Sigma Aldrich; Merck KGaA, Darmstadt, Germany). The lysates were incubated for 1 h at RT with shaking, subsequently sonicated 4 times for 30s and clarified by centrifugation at 15,000× *g* for 30 min at 4 °C. The supernatant was then filtrated using 0.22 µM filter units (Merck Millipore; Merck KGaA, Darmstadt, Germany) and the pH was adjusted to 7.4 with 10X TBS (0.5 M Tris, 1.5 M NaCl). FLAG-purification was performed using Anti-FLAG M2 Affinity Gel (Sigma Aldrich; Merck KGaA, Darmstadt, Germany), following the manufacturer’s instructions. Proteins were eluted with 0.1 M Glycine (pH 3.5) and collected in 1 M Tris (pH 8.0). The purification cycle was repeated several times and finally, all protein-containing eluates were pooled and dialyzed overnight against a 500-fold excess of 1X TBS (50 mM Tris-HCL, 150 mM NaCl; pH 7.4). Aliquots were stored at −80 °C until further use. 

### 2.6. Expression and Purification of SpyT Antigens

#### 2.6.1. Drosophila *S2* Cells

*S2* cells were grown in Insect-Xpress medium (Lonza, Köln, Germany). Adherent cultures were transfected with the respective pMT/BiP expression plasmids and pCoBlast (Invitrogen; ThermoFisher Scientifc, Dreieich, Germany) in a ratio of 20:1 using Effectene Transfection Reagent (#301425, Qiagen, Hilden, Germany) according to the instructions of the manufacturer. After transfection for 48 h 30 µg/mL Blasticidin (Invivogen, San Diego, CA, USA) was added to select stable polyclonal cell lines. For the SBV-GcH domain, cell cultures were expanded to a volume of 1.2 L (3 × 400 mL) in shaking flasks and incubated at +28 °C, 80 rpm. After 5 days each suspension culture was topped to 3 L and protein expression was induced with a final concentration of 2.5 µM CdCl_2_. Supernatants were harvested 8 days after induction by centrifugation and were subsequently concentrated to about 50 mL using a Vivaflow200 device (5000 MWCO PES; Sartorius, Göttingen, Germany). Biotin was blocked by addition of BioLock (iba lifesciences, Göttingen, Germany) as recommended. The concentrated supernatant was then purified using Streptactin-Superflow high capacity slurry (iba lifesciences, Göttingen, Germany) according to the manufacturer´s protocol. All protein-containing eluates were pooled, aliquoted and stored at −80 °C until further use. C-terminal Strep-Tags were not removed from the final constructs. SBV Gc core, AKAV-GcH and the SBV Gc stalk 1 domain were expressed and purified as described above but in smaller volumes (700–800 mL).

#### 2.6.2. Generation of the Fungal C1 Production Strain

The two Gc head-stalk expression vectors pMYT0451 and pMYT0452 were cut with *Pme*I and transformed together into the C1 production strain DNL131, from which nine protease genes had been deleted (unpublished) resulting in the SBV production strain DNL139. For transformation, the protoplast/PEG method was used [40] and the transformants were subsequently selected for hygromycin resistance. For this purpose, they were grown on selective medium in 24-well plate cultures. Supernatants were screened by Western blot analysis.

#### 2.6.3. C1 Fermentation and Purification of GcHS Antigen

For production of the GcHS antigen, the expression constructs were transformed in a *Thermothelomyces heterothallica* C1 production strain from which nine protease genes had been deleted (unpublished). The finally selected C1 strain producing GcHS was grown in a 1 L bioreactor in a fed-batch process with medium containing glucose (source of carbon) and (NH_4_)_2_SO_4_ and yeast extract (nitrogen sources). The production fermentation was verified by WB analysis (Figure S1) and carried out for 6 days at pH 6.8. The temperature was +38 °C in the batch phase and +25 °C in the feed phase. The GcHS antigen was harvested after 146 h of fermentation and purified by C-tag affinity chromatography with the CaptureSelect C-tag resin (ThermoFisher Scientific, Dreieich, Germany) according to the manufacturer’s protocols. The eluted product was dialyzed against PBS (10 mM Na_2_PO_4_, 138 mM NaCl, 2.7 mM KCl, pH 7.4).

### 2.7. Electron Microscopy Imaging

For transmission electron microscopy (TEM), samples were transferred to formvar coated TEM grids (400 mesh, Plano GmbH, Wetzlar, Germany) and stained with 2% phosphotungstic acid at pH 6.0. The grids were analyzed with a Tecnai-Spirit (FEI, Eindhoven, The Netherlands) at an accelerating voltage of 80 kV.

### 2.8. SDS-PAGE

SDS-PAGE was performed using a Mini-PROTEAN^®^Tetra System (Bio-Rad, Feldkirchen, Germany). Samples were mixed 1:1 with 2X SDS-Sample buffer, heated to 95 °C for 5 min and subsequently run on 12% Tris/Glycine SDS-Gels. InstantBlue (Expedon; 4basebio, Heidelberg, Germany) was used for Coomassie staining.

### 2.9. Antigen—LS Conjugation Reactions

All conjugation reactions were performed in 20 mM Tris-HCl, 300 mM NaCl, 0.2% Tween at pH 7.5. Volumes of reaction partners were calculated according to the selected molar ratio of antigen::SpyC-subunits. The reactions were incubated for 24–48 h at RT and subsequently kept at 4 °C until further use.

### 2.10. Generation of Vaccine Candidates for Animal Trials

(i) For the unsaturated LS-GcH (GcH displayed on LS with a conjugation efficiency of 10–50%) GcH and SpyC-LS were incubated in a molar ratio of 1::1.5 (antigen::LS subunit) in conjugation buffer for 48 h at RT without further processing.

(ii) For saturated LS-GcH particles (GcH displayed on LS with a maximum conjugation efficiency) a molar ratio of 3::1 was used and the conjugation product was subsequently purified by excessive dialysis against conjugation buffer until >90% of the unconjugated antigens were removed from the reactions.

(iii) For LS-GcHS, the GcHS domain was conjugated to SpyC-LS in a molar ratio of 1::3 for 48 h at RT.

For all preparations, conjugation efficiency and the final amount of bound antigen were determined by Western Blot (WB) and densitometric analysis using the ImageJ software (version 1.48; NIH). The indicated amounts of conjugated subunits refer to their total amount present in the reaction mixture.

The LS-Pept2 MPSPs were FLAG-purified and dialyzed against 1X TBS.

All immunogens were freshly prepared and quantified before immunization. Vaccine doses for mice were adjusted to 20 µg antigen/mouse in a total volume of 100 µL. Adjuvanted doses were supplemented with 10% (*v*/*v*) EMULSIGEN^®^ (MVP adjuvants, Omaha, NE, USA). 

For cattle, doses were adjusted to 50 µg of antigen in 1 mL total volume and supplemented with 10% (*v*/*v*) POLYGEN™ (MVP adjuvants, Omaha; NE). An overview of vaccines and experimental groups is given in Table S1.

### 2.11. Mouse Immunization-Challenge Trials

For all mouse trials, IFNAR-/- mice of the C57BL/6 genetic background (B6.129S2-Ifnar1tm1Agt/Mmjax) between 2 and 17 months of age were obtained from the specific pathogen-free breeding unit of the Friedrich-Loeffler-Institut, Greifswald-Insel Riems, Germany). For each trial, male and female as well as younger or older animals were distributed equally over groups of 9 animals each. Two additional mice served as non-treated environmental controls in each trial. Mice were immunized subcutaneously (s.c.) either once or twice 14 days apart. 21 days after the last immunization they were challenge-infected subcutaneously (s.c.) with 10^4^ or 10^6.4^ TCID_50_ per mouse of SBV strain BH619/12 (originally isolated 2012 [46]). Animals in mock groups were not vaccinated prior to challenge infection. Following challenge infection, the mice were weighed daily for 9 consecutive days and assessed for clinical signs. On days 3 and 7 post infection (pi), EDTA-blood samples were collected for RT-qPCR analysis. Mice showing severe clinical signs of disease were euthanized immediately; all surviving animals were sacrificed 21 days post infection. At necropsy, EDTA blood and serum as well as spleen and liver samples were obtained for RT-qPCR analyses.

### 2.12. Cattle Immunization Trial

16 cattle of a German domestic breed between 4 and 5 months of age were randomly assigned to 4 groups of 4 animals each. They were vaccinated s.c. twice 14 days apart, and 21 days after the 2nd immunization they were challenge-infected with 1 mL of a cattle-passaged SBV field strain [41]. Blood samples for serological analysis were collected at weekly intervals and on a daily basis for 10 consecutive days after challenge infection. All animals were euthanized 28 days after challenge infection. At necropsy, samples of spleen, tonsils, mandibular and mesenterial lymph nodes were obtained for RT-qPCR analyses.

### 2.13. Serology

Virus neutralization tests were performed in 96-well microtiter plates against SBV BH80/11-4 as described previously [47].

A commercially available N-protein-based ELISA (ID Screen Schmallenberg virus Competition Multi-Species, IDvet) was used for detection of SBV N-specific antibodies.

In-house ELISAs to assess the functionality of peptide epitopes as well as GcH and GcHS antigens: ELISA plates (medium-binding; Greiner, Frickenhausen, Germany) were coated overnight at 4 °C with 100 ng/well of the respective antigen in 0.1 M carbonate buffer. The plates were blocked with 5% skimmed milk for 1 h at 37 °C. Subsequently, sera pre-diluted in PBS + 0.05% Tween20 were added for 1 h at 37 °C. For detection, the following HRP-conjugated secondary antibodies were incubated for 1 h at RT: anti-bovine HRP (1:20,000; A5295-1ML, Sigma Aldrich; Merck KGaA, Darmstadt, Germany), anti-ovine HRP (1:10,000; 313-036-003, dianova, Hamburg, Germany) or anti-mouse HRP (1:1000; 115-036-003; dianova). Between each incubation step, the plates were washed three times with PBS + 0.05% Tween. 

All field serum samples were provided by the National Reference Laboratory for SBV (Friedrich-Loeffler Institut, Greifswald, Insel Riems, Germany). Additional sera were obtained from previously performed animal trials [37,48,49]. 

### 2.14. RNA-Extraction and RT-qPCR

RNA extraction from EDTA-blood, serum and tissues samples was performed on a KingFisher 96 Flex instrument (ThermoFisher Scientific, Dreieich, Germany) using the NucleoMag^®^VET kit (MACHERY-NAGEL, Düren, Germany) according to the manufacturer’s instructions. For SBV RNA detection a previously described S segment-based RT-qPCR assay was applied with an external standard for viral RNA quantification [50]. All RT-qPCR analyses were executed on a BioRad CFX96 Touch real-time PCR detection system (Bio-Rad, Feldkirchen, Germany).

### 2.15. Statistical Analysis

All analyses were performed using GraphPad Prism version 8.4.2. The non-parametric Kruskal–Wallis test was applied for the comparison of more than two groups and Dunn’s test was used for subsequent multiple comparisons between individual groups. *p* values < 0.05 were considered as significant. For analysis of survival curves, the Mantel–Cox test was used. 

## 3. Results

### 3.1. Selection of Model Antigens

Based on previous experimental data and structural analyses that confirmed their immunogenicity [37,38], the SBV Gc head domain (GcH, aa 465–702) and the Gc head-stalk domain (GcHS, aa 465–874) were selected as model antigens to test the functionality of the plug-and-display approach (Figure 1).

To identify linear epitopes, HiSense Pepscan analysis was performed using the SBV Gc ectodomain synthesized as a library of overlapping peptides. The library was probed with sera from SBV-infected cattle or sheep and three candidate epitopes could be detected (Table 1). The respective peptides (#1–3) were fused to LS and evaluated for their ELISA reactivity with SBV-specific antibodies. The reactivity of peptide #2 (QTLTTLSLIKGAHRN, aa 694–708) was not significantly different from that of the full-length control antigen and reached higher OD values in ELISAs than peptides #1 and #3. We therefore selected LS-Pept2 as the vaccine candidate for subsequent in vitro and in vivo experiments (Figure S2).

### 3.2. Expression and Purification of LS Scaffold and SpyT-Antigens

The N-terminal fusion of the SpyC sequence did not interfere with the self-assembly of the LS and particles of 15 nm-size could be visualized by electron microscopy in FLAG-tag purified preparations. 

For the recombinant GcH antigen produced in Drosophila *S2* cells, structural integrity and antigenicity was analyzed and confirmed in a previous study [38]. 

The GcHS antigen was successfully expressed in the newly generated C1-production strain as described in Materials & Methods. The secreted recombinant proteins were purified using C-tag affinity chromatography with a final yield of 1.83 g protein per 1 L culture supernatant. The antigenicity of the purified protein in comparison to the *S2*-produced GcH was controlled by ELISA using sera from experimentally or field-infected cattle and sheep (Figure S3).

### 3.3. Plug-and-Display Platform

#### 3.3.1. Conjugation Efficiency

**Figure 2:**
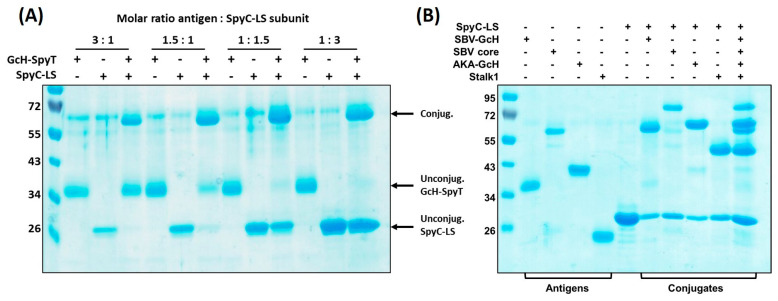
Versatility of the Plug-and-display LS platform. (**A**) Conjugation efficiency is dependent on the molar ratio of antigen input vs. provided SpyC-LS subunits. Conjugation reactions were performed using molar ratios of 3:1; 1.5:1; 1:1.5 and 1:3 (antigen:SpyC-LS subunits). Reducing SDS-PAGE and Coomassie staining was performed after incubation for 48 h at RT. Input of SpyT-antigen and SpyC-LS are loaded next to the lanes showing the conjugation products; (**B**) Different antigens can be efficiently conjugated. Conjugation reactions were performed with SpyT-equipped SBV-GcH, SBV Gc core, SBV stalk1 and AKAV-GcH. All antigens were either conjugated separately with SpyC-LS or all together in one single reaction. Reducing SDS PAGE and Coomassie staining was performed after 48 h incubation at RT.

We observed that the conjugation efficiency, i.e., the amount of antigen-bound LS-SpyC-subunits, is mainly dependent on the molar ratio of the two provided reaction partners (Figure 2A). Using a 3-fold molar excess of SpyT-antigen allowed for conjugating the maximal number of antigens fitting on the LS surface (saturated particles). However, in this approach the amount of effectively conjugated antigen has to be determined by densitometric analysis since about 30–50% remain unconjugated and have to be removed from the final reaction product by dialysis. In contrast, the production of particles with a low conjugation efficiency (10–50% of SpyC-subunits conjugated) promotes binding of all provided antigens obviating a final purification step. This strategy required a 1.5 to 3-fold excess of LS-SpyC subunits compared to antigens. Since here, the amount of conjugated antigen equals the input, a subsequent quantification by WB is not necessary.

#### 3.3.2. Versatility 

**Figure 3:**
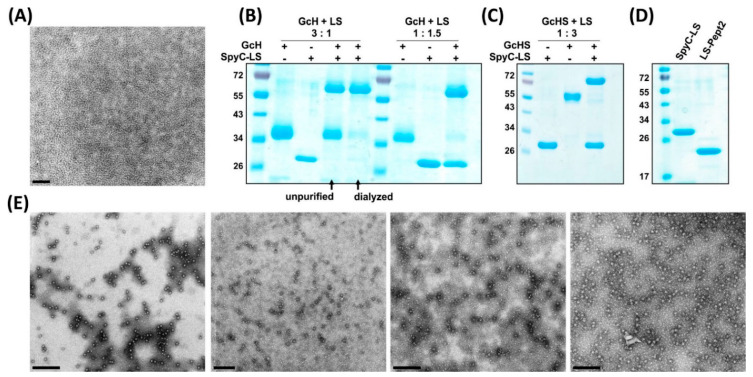
Final vaccine candidates. (**A**) Negatively stained TEM of the SpyC-LS MPSP. (**B**) Reducing SDS-PAGE and Coomassie staining showing input partners and the respective conjugation products of the saturated (antigen:LS subunit = 3:1) and unsaturated (1:1.5) LS-GcH MPSP preparations used for immunizations. Saturated LS-GcH particles were purified by dialysis in order to remove unconjugated GcH monomers as indicated by arrows. (**C**) Reducing SDS-PAGE and Coomassie staining showing input partners next to the conjugation product of the final LS-GcHS (antigen:LS subunits = 1:3) MPSP vaccine. (**D**) Reducing SDS-PAGE and Coomassie staining of the LS-MPSP after N-terminal genetic fusion of the peptide epitope #2 (LS-Pept2) in comparison to the SpyC-LS backbone. (**E**) TEM images of the finally selected vaccine candidates for evaluation in the target species. From left to right: LS-Pept2, LS-GcH saturated, LS-GcH unsaturated, LS-GcHS. Scale bars 200 nm.

We investigated the suitability of the SpyC-LS for displaying different antigens independently of their size by conjugating a panel of SpyT-fused proteins. Although the LS-scaffold is of rather small size (about 15 nm, Figure 3A), all constructs could be successfully conjugated. We observed that the conjugation efficiency may have been influenced by the actual size of the antigen since the coupling of larger-size constructs (>50 kDa) was somewhat less efficient, possibly due to sterical hinderance. Nonetheless, it was possible to conjugate several antigens in the same reaction. Choosing appropriate molar ratios for each antigen compared to the SpyC-subunits allowed them all to be bound with a similar efficiency (Figure 2B). This affirms the versatility of the SpyC-LS scaffold and its suitability as a modular vaccine platform, especially when used in combination with the protein superglue system.

### 3.4. Generation of Vaccine Candidates

In order to assess the functionality and applicability of the LS MPSP in vivo, we generated four vaccine candidates for immunogenicity trials in a small animal model as well as in cattle, an important target species of SBV.

LS-GcH MPSPs were designed to investigate the efficacy of the LS-conjugated GcH compared to the monomeric GcH. In addition, we aimed to assess the potential influence of the conjugation efficiency on the immune response and therefore generated two preparations of LS-GcH MPSPs:

(i) Saturated LS-GcH: Conjugation reactions were performed using a molar ratio of 3::1 (antigen::SpyC-subunit). After incubation for 48 h at RT, about 95% of all SpyC-subunits detectable by WB were bound to SpyT-antigens. The amount of unconjugated antigen in the final reaction product was estimated to about 36% by densitometry and was subsequently reduced to <4% by excessive dialysis (Figure 3B).

(ii) Unsaturated LS-GcH: Conjugation reactions were performed using a molar ratio of 1::1.5 (antigen::SpyC-subunits). After 48 h incubation at RT, about 96% of the provided antigens that could be detected by WB-staining with an anti-Gc antibody were bound to SpyC-subunits with an estimated 40% conjugation efficiency (Figure 3B).

To confirm the versatility of the LS MPSP and its suitability to present antigens expressed in different non-mammalian hosts we produced particles displaying the GcHS antigen produced in the C1 fungal system:

(iii) LS-GcHS (unsaturated): Here, SpyC-LS subunits were used in a 3-fold molar excess compared to the SpyT-GcHS antigen. After 48 h incubation at RT about 98% of GcHS was conjugated occupying 33% of the LS-SpyC coupling sites (Figure 3C).

We further aimed to directly compare the plug-and-display strategy to the presentation of the linear peptide #2 epitope directly fused to LS: 

(iv) LS-Pept2 was expressed in *E. coli* and purified by FLAG affinity chromatography (Figure 3D).

### 3.5. Evaluation of Vaccine Candidates in an IFNAR-/- Mouse Infection Model for SBV 

#### 3.5.1. Impact of a MPSP-Antigen Display on Immune Response and Vaccine Efficacy

**Figure 4:**
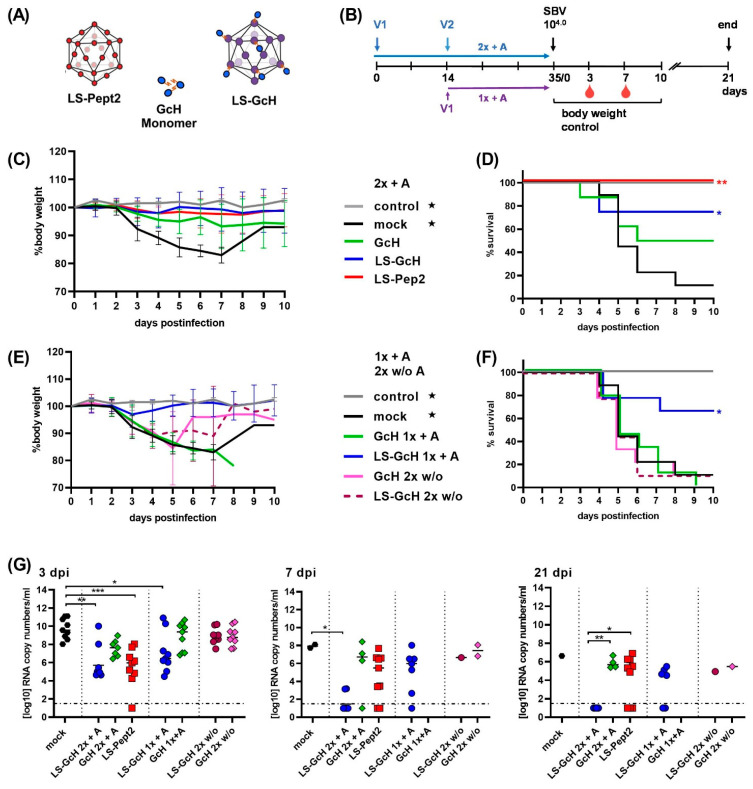
IFNAR-/- mouse trial #1. Efficacy of the LS-conjugated GcH domain compared to the monomeric GcH and the LS-presented peptide epitope QTLTTLSLIKGAHRN. (**A**) Schematic of vaccines used for the immunizations in this trial; (**B**) Experimental scheduling. V1 and V2 indicate time-points of the 1st and 2nd vaccination; (**C**) Body weight development after challenge infection in groups vaccinated twice with adjuvanted vaccines (2x + A). Each line represents the mean value of the respective group with standard deviation (SD); (**D**) Survival curves in groups vaccinated 2x + A; (**E**) Body weight development after challenge infection in groups vaccinated once with adjuvanted vaccines (1x + A) or twice in the absence of adjuvant (2x w/o A); (**F**) Survival curves in groups vaccinated 1x A or 2x w/o A; For (**C**,**E**) as well as for (**D**,**F**) data of the respective control and mock groups (marked with stars) were inserted in both graphs; (**G**) SBV RNA detected by RT-qPCR in EDTA blood samples of surviving animals in each group at 3, 7 or 21 dpi, respectively. Dashed lines indicate the detection limit of the RT-qPCR assay. Samples of animals that succumbed to infection or had to be euthanized prior to or on the respective sampling day were not included. In (**G**) statistical analysis was performed using the Kruskal–Wallis test followed by Dunn´s test for comparisons between individual groups. *p* values < 0.05 were considered significant. (* *p* < 0.05; ** *p* < 0.01; *** *p* < 0.001). Only significant differences between groups are labeled. Differences that are not significant (*p* > 0.05) are not separately indicated. In (**D**,**F**) significant differences compared to the mock control were calculated using the Mantel–Cox test (* 0.0332; ** 0.0021; *** 0.0002; **** <0.0001). Comparisons between all groups against each other are not indicated but are shown in Table S2.

Groups of 9 mice were vaccinated once or twice with the monomeric GcH, LS-GcH MPSPs or LS-Pept2 formulated either with or without adjuvant (Figure 4A,B). Three weeks after the last vaccination, the animals were challenge-infected with SBV strain BH619 with the standard dose of 10^4^ TCID_50_/mouse.

8 out of 9 mock-vaccinated animals died or had to be euthanized within 5 days pi, confirming a successful infection. After prime-boost administration in the presence of adjuvants, 6/8 animals vaccinated with LS-GcH and 4/8 receiving the monomeric GcH remained healthy (Figure 4C,D). Without boosting, the monomer failed to induce a protective immune response, whereas the LS-conjugated GcH protected 6/9 animals from clinical disease (Figure 4E,F). Thus, a multimeric presentation on the LS-scaffold markedly improved vaccine efficacy. Interestingly, this adjuvanting effect was also evident for the LS-presented peptide #2 since all mice vaccinated with this construct remained completely healthy after challenge infection (Figure 4C,D). However, without the addition of an adjuvant, none of the particulate vaccines were sufficiently immunogenic to confer protection (Figure 4E,F). 

All surviving animals in each group showed neither any clinical signs of disease, nor loss of body weight. Nevertheless, in all mice high levels of SBV genome equivalents were detected by RT-qPCR on days 3 and 7 pi (Figure 4G). Animals vaccinated once or twice with Emulsigen-admixed LS-GcH or with LS-Pept2 showed significantly reduced RNA copy loads compared to the mock control. All surviving animals in the twice-vaccinated LS-GcH group were able to clear viral RNA until the end of the trial at 21 dpi, which again supports the superior performance of the LS-conjugated antigens.

#### 3.5.2. Influence of Conjugation Efficiency on Vaccine Performance

**Figure 5:**
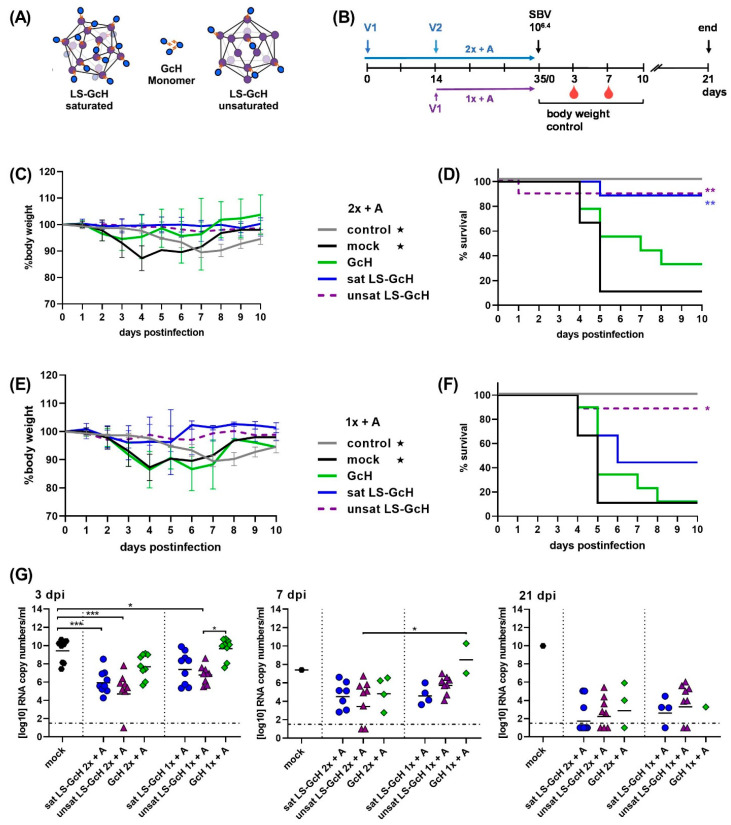
IFNAR-/- mouse trial #2. Efficacy of saturated and unsaturated LS-GcH MPSPs in comparison to monomeric GcH. (**A**) Schematic of vaccines used for the immunizations in this trial; (**B**) Experimental scheduling. V1 and V2 indicate the time-points of the 1st and 2nd vaccination; (**C**) Body weight development after challenge infection in groups vaccinated twice with adjuvanted vaccines (2x + A). Each line represents he mean value of the respective group with SD; (**D**) Survival curves in groups vaccinated 2x + A; (**E**) Body weight development after challenge infection in groups vaccinated once with adjuvanted vaccines (1x + A); (**F**) Survival curves in groups vaccinated 1x + A; For (**C**,**E**) as well as for (**D**,**F**) data of the respective control and mock groups (marked with stars) were inserted in both graphs; (**G**) SBV RNA detected by RT-qPCR in EDTA blood samples of surviving animals in each group at 3, 7 or 21 dpi, respectively. Dashed lines indicate the detection limit of the RT-qPCR assay. Samples of animals that succumbed to infection or had to be euthanized prior to or on the respective sampling day were not included. In (**G**) Statistical analysis was performed using the Kruskal–Wallis test followed by Dunn´s test for comparisons between individual groups. *p* values < 0.05 were considered significant. (* *p* < 0.05; ** *p* < 0.01; *** *p* < 0.001). Only significant differences between groups are labeled. Differences that are not significant (*p* > 0.05) are not separately indicated. In (**D**,**F**) significant differences compared to the mock control were calculated using the Mantel–Cox test (* 0.0332; ** 0.0021; *** 0.0002; **** <0.0001). Comparisons between all groups against each other are not indicated but are shown in Table S2.

In a second trial we aimed to investigate if saturated and unsaturated particles might stimulate divergent immune responses. Therefore, we directly compared the performance of particles with either a high (about 95%; sat LS-GcH) or a low (about 40%; unsat LS-GcH) conjugation efficiency, respectively (Figure 5A). Unconjugated antigens were removed from the saturated LS-GcH preparation by dialysis in order to compare the same amounts of conjugated antigens in each vaccine.

Groups of 9 mice each were then vaccinated with either monomeric GcH, saturated LS-GcH or unsaturated LS-GcH MPSPs (Figure 5B). Notably, in this trial we accidentally used a very high challenge dose (10^6.4^ TCID_50_/mouse). 

In both groups vaccinated twice with the two different LS-GcH preparations, 8/9 animals survived challenge infection without any clinical signs of disease. A single shot vaccination with unsaturated LS-GcH still protected 8/9 animals compared to 4/9 in the saturated LS-GcH group. Monomeric GcH conferred protection in 4 out of 9 mice after prime-boost delivery. However, without boosting only one animal survived challenge infection. This confirms the previously observed improved immunogenicity of LS-conjugated GcH, but also the need for a booster vaccination (Figure 5C–F). 

Again, all animals became viremic after challenge infection, with high loads of SBV RNA detected in blood samples. Thus, clinically protected animals were not able to prevent replication of challenge virus. Nevertheless, viral copy loads in mice vaccinated twice with the LS-conjugated GcH preparations were significantly lower than in the mock-controls. For unsaturated particles a significant reduction could be achieved even after a single immunization (Figure 5G). In this trial, the majority of mice were not able to clear the challenge virus because of the 100x-fold increased challenge dose compared to the first experiment.

As already shown in the previous study, the MPSP-antigen display clearly improved the immunogenicity of the GcH antigen. Based on our experimental data, we assume that the different survival rates observed between saturated and unsaturated LS-GcH vaccines were caused by statistical variations between sampling groups rather than by real differences in efficacy. Thus, an increase in antigen density on each particle does not significantly affect the quality of the immune response.

#### 3.5.3. Evaluation of the GcHS Antigen Produced in C1

In a third trial we addressed the functionality of the C1-produced GcHS protein and whether a conjugation to LS can improve its immunogenicity.

**Figure 6:**
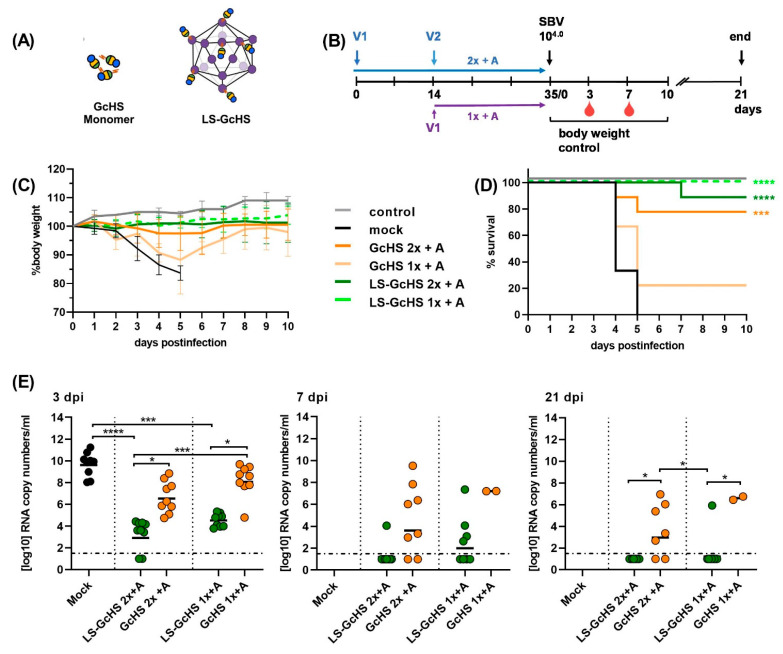
IFNAR-/- mouse trial #3. Evaluation of the C1-produced GcHS domain and the protective efficacy of the monomeric and LS-conjugated antigen. (**A**) Schematic of vaccines used for the immunizations in this trial; (**B**) Experimental scheduling; (**C**) Body weight development after challenge infection, each line represents the mean value of the respective group with SD; (**D**) Survival curves after challenge infection. One animal in group LS-GcHS vaccinated twice (2x + A) accidentally died during blood sampling in a manner unrelated to SBV infection; (**E**) SBV RNA detected by RT-qPCR in EDTA blood samples of surviving animals in each group at 3, 7 or 21 dpi, respectively. Dashed lines indicate the detection limit of the RT-qPCR assay. Samples of animals that succumbed to infection or had to be euthanized prior to or on the respective sampling day were not included. In (**E**) statistical analysis was performed using the Kruskal–Wallis test followed by Dunn´s test for comparisons between individual groups. *p* values < 0.05 were considered significant (* *p* < 0.05; ** *p* < 0.01; *** *p* < 0.001; **** *p* < 0.0001). Only significant differences between groups are labeled. Differences that are not significant (*p* > 0.05) are not separately indicated. In (**D**) significant differences compared to the mock control were calculated using the Mantel–Cox test (* 0.0332; ** 0.0021; *** 0.0002; **** <0.0001). Comparisons between all groups against each other are not indicated but are shown in Table S2.

We therefore immunized groups of 9 mice with the monomeric GcHS or with unsaturated LS-GcHS MPSPs (LS-GcHS). In order to allow a direct comparison to the first trial, challenge infection was performed again with the standard dose of 10^4^ TCID_50_/mice of SBV strain BH619 (Figure 6A,B).

All mock-vaccinated mice died or had to be euthanized within 5 days pi. The GcHS subunit protected 7/9 animals vaccinated twice. Without boosting, only 2 out of 9 mice survived the challenge infection. In contrast, LS-GcHS conferred complete protection with and without a booster immunization (Figure 6C,D). As observed before, replication of challenge virus was not blocked in protected animals and SBV RNA was detected in all blood samples collected after challenge infection. However, the LS-GcHS-vaccinated groups displayed significantly lower viral copy numbers than mock- and subunit-vaccinated animals (Figure 6E). Two animals vaccinated with MPSP-displayed GcHS-antigen remained even RT-qPCR-negative and did not seroconvert for N-specific antibodies, which probably indicates sterile immunity. Except for one animal receiving only a single vaccination, all LS-GcHS-vaccinated mice had cleared the infection at the end of the trial. Taken together, the C1-expressed recombinant GcHS is fully immunogenic and its vaccine efficiency can be markedly improved by presentation on the multimeric LS scaffold. 

### 3.6. Evaluation of Selected LS-MPSP Vaccine Candidates in Target Species

**Figure 7:**
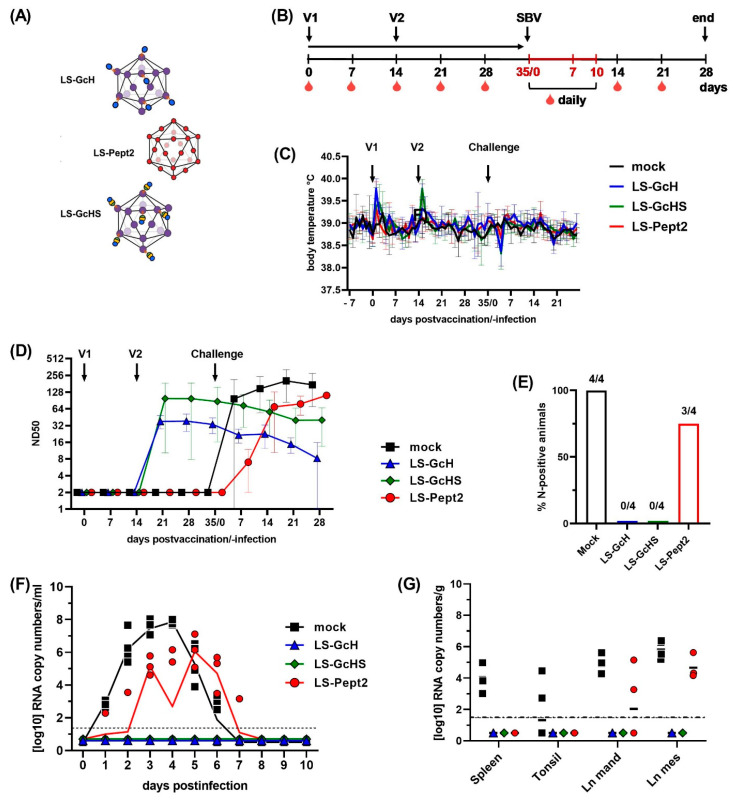
Evaluation of finally selected vaccine candidates in cattle. (**A**) Schematic illustration of the final candidate vaccines used in the trial; (**B**) Vaccination and sampling schedule, V1 and V2 indicate time-points of the 1st and 2nd immunization; (**C**) Mean body temperatures after vaccination and challenge infection. Each line represents the mean value of the respective groups with SD; (**D**) Development of neutralizing antibodies after immunizations and challenge infection, determined by VNT. Mean values and SD of ND_50_ titers are indicated for each group; (**E**) Detection of SBV-Nucleoprotein-specific antibodies by ELISA in serum samples collected on 21 dpi; (**F**) SBV genome equivalents in serum samples that were collected daily for 10 days after challenge infection. Geometric means are indicated by connected solid lines; (**G**) SBV RNA detected in tissue samples of each animal collected at necropsy 28 dpi. In (**F**,**G**) dashed lines indicate the detection limit of the RT-qPCR assay. In (**D**–**G**) one animal vaccinated with LS-Pept2 was excluded from data analysis, since it showed no reaction to either immunization or challenge infection. No SBV RNA was found in any sample tested by RT-qPCR and the animal did not develop antibodies towards the LS-Pept2 antigen and neither neutralizing nor N-specific antibodies after challenge infection.

We finally tested the most promising LS-MPSP vaccine candidates, namely LS-GcH, LS-GcHS and LS-Pept2, in cattle, a main target species of SBV (Figure 7A). For the LS-GcH and LS-GcHS MPSPs used in this trial, we prepared unsaturated particles with conjugation efficiencies of about 40% and 30%, respectively. Three groups of 4 calves each were vaccinated two times 14 days apart. Three weeks after boosting, all animals were inoculated s.c. with 1 mL of infectious SBV-positive serum (Figure 7B). 

One day after both prime and boost vaccination, all animals showed an increase in body temperatures for one day (Figure 7C). We cannot exclude that this is attributable to residual endotoxins in the particle preparations. However, this was not further investigated. Apart from that, no adverse reactions or clinical signs of disease could be observed in any group throughout the whole trial. 

One week after the second immunization (day 21), groups vaccinated with LS-GcH or LS-GcHS developed high titers (50% neutralization dose ND_50_ from 24 to 229) of neutralizing antibodies with maximum levels reached on day 28 (Figure 7D). No further increase was observed after challenge infection and no SBV-N-specific antibodies could be detected until the end of the trial (Figure 7E). In accordance with this serological data, none of the animals scored positive for SBV RNA by RT-qPCR at any sampling time-point (Figure 7F). Furthermore, all tissue samples collected at necropsy 28 days pi tested negative for SBV genomes (Figure 7G), confirming complete protection and a robust sterile immunity. 

In contrast, mock- and LS-Pept2-vaccinated animals did not develop any detectable neutralizing antibodies prior to challenge infection and all animals seroconverted for N-specific antibodies following challenge infection (Figure 7D,E). Thus, in both groups replication of the challenge virus was not prevented, which was further confirmed by RT-qPCR detection of SBV RNA in serum samples starting from day 1 or 2 pi for 5 to 6 consecutive days. (Figure 7F). In addition, SBV genomes could still be detected in tissue samples obtained at necropsy (Figure 7G).

## 4. Discussion

Here, we present the development of a modular vaccine platform based on lumazine synthase from *Aquifex aeolicus* and experimental data demonstrating the versatility and efficacy of this novel multimeric protein scaffold particle platform, both in a small animal model and in target species.

In general, the key to the development of efficacious vaccines relies on the identification of a suitable protective antigen. In the case of newly emerging pathogens it is therefore essential to rapidly identify key immunogens. For these purposes, a large number of bioinformatic and -omics tools are these days freely available and enable in silico predictions in a relatively short time frame [48,49]. However, *de novo* designed epitope-based vaccines seldomly induce appropriate humoral immune responses, since the epitopes are generally not presented in their native conformation [51,52]. 

We performed Pepscan analysis using sera from animals that had recovered from an SBV infection, with the aim of identifying epitopes that are recognized by naturally produced antibodies. In mice, our candidate peptide #2 fused to LS (LS-Pept2) conferred protection from an otherwise lethal challenge dose in all of the vaccinated animals. However, it failed to induce a protective immune response in cattle, a main target species of SBV. Based on the previously resolved crystal structure of the SBV spike, the peptide epitope is located at the suggested flexible interface between the Gc head and stalk domain [38]. Thus, it can be presented in different conformations at the virion surface. In contrast, the LS-fused peptide is displayed only in one specific (and potentially artificial) orientation. Thus, antibodies induced after vaccination might not be able to access the native epitope and consequently lack neutralizing capacity. In order to optimize the efficacy of the LS-peptide vaccines, all LS-fused epitopes detected by Pepscan analysis could be applied together as a cocktail to induce a broader antibody response and to improve immunogenicity in target species. The discordant, superior performance of the LS-Pept2 vaccine in the IFNAR-/- mouse model most likely reflects the species-specific diversity in B-cell and antibody repertoires [53]. This finding further underscores the value of investigating the efficacy of candidate vaccines in target species early in vaccine development.

However, neutralizing antibodies often target conformational epitopes or antigenic domains and identifying such key immunogens is complex and time-consuming. In addition, the respective antigens must be produced in host systems providing the post-translational modifications required for proper folding of the antigen. Since we have previously shown that the antigenicity of SBV GcH and GcHS depends on their correct conformation [37,38,54] we used here a plug-and-display strategy to bind the two separately expressed antigens to the pre-fabricated LS MPSP. We deliberately focused on non-mammalian expression hosts since they are attributed with a reduced risk of mammalian viral contaminants. This would be beneficial with regard to a fast track regulatory process and an accelerated licensing procedure of candidate vaccines. 

The insect *Drosophila S2* cells provide an eukaryotic environment as well as the majority of post-translational modifications found in mammalian cells [55]. We have previously shown that the *S2*-expressed SBV GcH domain is fully functional and can induce protective antibodies upon vaccination. Thus, its immunogenicity is comparable to that of GcH expressed in mammalian HEK293T cells [37,38]. The GcHS domain was produced using the C1 fungal system in order to assess the potential of this technology to produce immunogenic proteins. The C1 technology was initially developed for large scale and high yield expression of industrial enzymes, but current efforts focus on the manufacturing of biologicals and drugs [40,56]. It has been shown that N-glycosylation in C1 enzymes does not include as large high-mannose glycan structures as found in *Aspergillus* species [54,57] or the hyperglycosylated N-glycan structures found in several yeast species. The C1-produced GcHS induced a highly protective immune response in both mice and cattle. Presented on LS-particles, it even conferred complete clinical protection after a single shot immunization in mice. Thus, this technology represents a promising strategy for a cost-effective, flexible and high-yield production of therapeutic recombinant proteins.

In accordance with previous studies [24], SpyC/SpyT-mediated conjugation reactions were easy to perform, highly stable and reproducible with antigens of varying size and nature. We additionally showed that several antigens can be conjugated in one single reaction with a similar efficiency. This feature could be exploited to elicit more broadly reacting antibodies, e.g., by a combined display of variable and more conserved domains [19]. A combined presentation of GcH domains from different orthobunyaviruses (e.g., Shuni virus, Akabane virus or Oropouche virus) might even induce antibodies directed against all of the admixed antigens and thereby provide virus-family-wide protection. 

It has already been demonstrated that a tetravalent dengue virus subunit vaccine stimulates an immune response against each serotype [58], and orthogonal display on IMX313 scaffolds improved the generation of antibodies against both of the presented proteins [23]. A co-delivery of additional immunostimulatory elements, e.g., T-cell epitopes on the same scaffold, represents another attractive feature for improving the quality and duration of the antibody response [13,59,60]. Even though, presentation of GcH and GcHS on the LS scaffold markedly improved their efficacy, protection was still dependent on the application of an adjuvant, as it has been reported repeatedly for non-infectious particulate vaccines [61,62]. 

We demonstrated that the conjugation level of different antigens to the LS is dependent on the molar ratio of antigen::LS-SpyC subunits supplied in the reactions. In the majority of similar studies, the antigen was provided in a 1.5- to 3-fold molar excess in order to conjugate the maximum amount of SpyC-subunits and to produce homogeneous particles [22,23,24,25,29,63]. However, in the context of newly emerging pathogens, the production process for each antigen has to be started from the beginning. Consequently, the generation of antigen-saturated particles that require very high amounts of recombinant proteins inevitably slows down the development process. In contrast, the bacterially expressed LS-scaffold can be easily produced at a large scale and stockpiled until use. The manufacturing of unsaturated particles therefore represents a cheaper and less time-consuming approach. It has been reported that even particles with a conjugation level of only 10% can raise an efficient antibody response after booster vaccination [26]. In order to verify these results for our vaccine candidates, we directly compared the potency of LS MPSPs with high and low antigen conjugation levels. In our studies, both preparations performed equally well and protected >80% of the vaccinated animals from clinical signs of disease even after a one-shot application. Thus, at least in the present work, an increase in the antigen density on the LS-scaffold was not beneficial with regard to immunogenicity. We hypothesize that the small size of the antigen-loaded particles already promotes B-cell receptor crosslinking, an efficient drainage to lymph nodes and a rapid uptake by antigen-presenting cells. Therefore, it may not be necessary to saturate MPSPs with proteins to elicit an optimal immune response. This is supported by the previous finding that an enhanced antigen concentration per particle affects vaccine efficacy only when the actual antigen dose is increased [64]. When using a platform for presentation of antigens to the immune system, it is relevant to consider if pre-existing immunity, or an immune response elicited by the scaffold upon (repeated) vaccination, could compromise vaccine efficacy. Since LS is derived from the hyperthermophilic bacterium *Aquifex aeolicus*, pre-existing immunity against the LS scaffold or the bacterial superglue components is unlikely to exist in animal or human populations. Interestingly, Howarth et al. recently showed that a potential impact of antibodies against components of SpyC and SpyT can be prevented by using the novel SpyC/SpyT 003 pair [30]. Nevertheless, it is presently unclear if immune responses elicited by LS upon vaccination could compromise the repeated use of this scaffold. Theoretically, an immune response against the scaffold elicited by a first vaccination could either have an adjuvanting effect (providing T-cell help) or could result in increased clearance. Previous studies found that anti-carrier antibodies can indeed affect the immune response against the presented antigen [59,65]. However, it was also demonstrated that high levels of antigen-specific antibodies can be induced despite a pre-existing carrier immune response [65]. In our study, animals vaccinated with LS-conjugated antigens developed varying amounts of anti-scaffold antibodies as was observed previously in a MERS-CoV model [60]. However, we could not observe obvious interference of the anti-LS antibodies with the immune response against the MPSP-displayed antigen. In mice, protection from clinical signs of disease could be improved using a prime-boost regimen, and in cattle, boosting had no impact on the development of high titers of neutralizing antibodies. Nevertheless, the effect of several booster applications will have to be evaluated in the future using different antigens displayed on the same LS-MPSP.

Even though the suitability of the IFNAR-/- mouse model for SBV vaccination-challenge studies is well established [66], it has to be considered that IFNAR-/- mice are not able to respond to type I interferons and are consequently highly susceptible to viral infections [67]. Since immunogenicity studies in this artificial model have to be assessed critically, we also tested our final vaccine candidates in immune-competent target animals to finally confirm their efficacy.

In cattle, LS-conjugated GcH and GcHS antigens induced high titers of neutralizing antibodies within 21 days of the first immunization and conferred sterile immunity, completely inhibiting replication of challenge virus. The capacity to induce such a rapid and sound immune response is an important quality in preventing the spread of an emerging pathogen and is remarkable, considering the non-infectious nature of the MPSP vaccines. Since the animals were vaccinated twice with a 14 day interval, it remains unknown if a single application would be also sufficient to elicit protective immunity. However, a one-shot immunization with the same LS-displayed antigens protected about 80% of IFNAR-/- mice from clinical signs of disease after inoculation with an otherwise lethal challenge dose. Using the cattle model, we demonstrated before that among inactivated SBV prototype vaccines [68], the SBV Gc head domain, either as a HEK293T-produced recombinant subunit or delivered by a Modified Vaccinia Ankara (MVA) vector [37,69], confers sterile immunity after a prime-and-boost application. Nevertheless, none of these previously tested vaccines induced a robust level of neutralizing antibodies prior to a booster vaccination. This further highlights the high potential of an antigen display on LS MPSPs to enhance and accelerate the onset of a protective immune response. Further animal studies will validate these results in more detail in the future.

## 5. Conclusions

Here, we present the development of a modular vaccine platform based on the lumazine synthase from *Aquifex aeolicus*. We demonstrate that the LS represents a versatile multimeric scaffold that is suitable for the display of directly fused linear epitopes as well as for large and complex antigens using a protein superglue plug-and-display strategy. We additionally show that peptide epitopes are inferior to antigenic domains with regard to immunogenicity and to the induction of protective neutralizing antibodies.

Our platform enables both a simple design as well as a reliable and reproducible production of vaccine candidates. We show that presenting antigens on the LS-MPSPs improves their immunogenicity and vaccine efficacy both in a small animal model as well as in target species. Thus, the presently tested platform represents a very promising tool for producing efficacious vaccines that could be available in a short time, which is of major importance especially in case of newly emerging and re-emerging infectious diseases.

## Figures and Tables

**Table 1:** Linear peptide epitopes detected by PepScan analysis.

Peptide #	aa Sequence	aa Position
#1	ASVDEQELIKSLNLN	508–522
#2	QTLTTLSLIKGAHRN	694–708
#3	TLSLIKGA	698–705

## Data Availability

All data supporting the conclusions of this study is contained within the article or within Supplementary Materials.

## Supplementary Materials

The following are available online at https://www.mdpi.com/article/10.3390/vaccines9060651/s1, Figure S1: Analysis of the C1 production fermentation by Western blot and Coomassie-staining, Figure S2: ELISA reactivity of peptide epitopes identified by Pepscan analysis, Figure S3: Comparative ELISA reactivity of the *S2*-produced SBV-GcH and the C1-produced GcHS domains with SBV-antibody positive or negative serum samples, Table S1: Summary of immunogenicity trials with an overview of vaccines, doses and animals, Table S2: Statistical analysis of survival curves shown in Figure 4D,F, Table S3: Statistical analysis of survival curves shown in Figure 5D,F, Table S4: Statistical analysis of survival curves shown in Figure 6D.
