# Supplementary material for: Development of a Modular Vaccine Platform for Multimeric Antigen Display Using an Orthobunyavirus Model
Source: Vaccines (Basel). 2021 Jun 15;9(6):651. doi: 10.3390/vaccines9060651 (PMC8232151; doi:10.3390/vaccines9060651)
Supplement: Supplementary file 1 [file vaccines-09-00651-s001.zip › vaccines-1230291-supplementary.pdf]

vaccines

MDPI

Supplementary materials

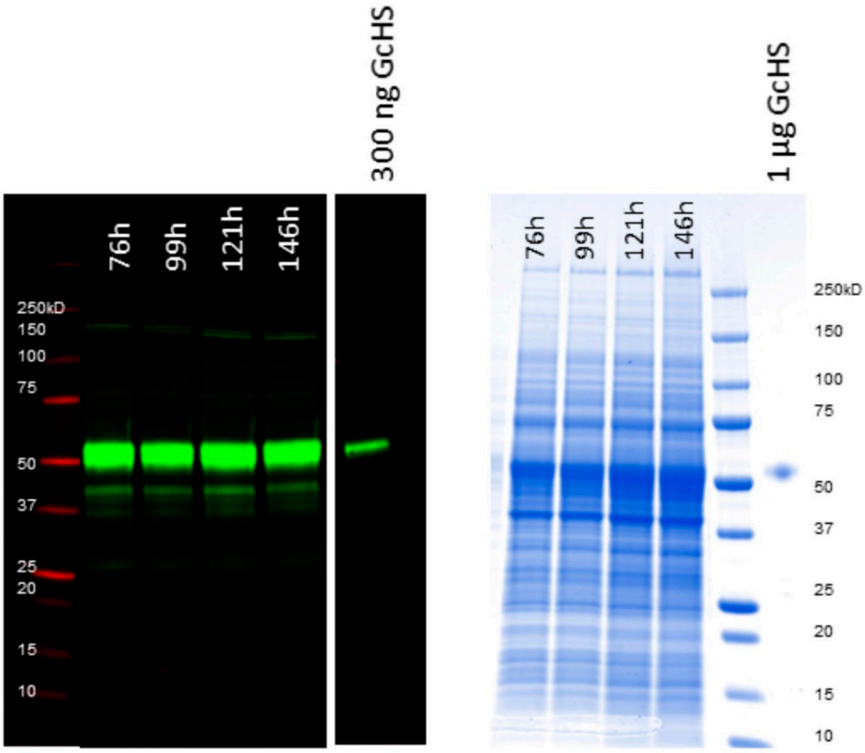

Figure S1. Analysis of the C1 production fermentation by Westernblot (left) and Coomassie-staining (right). The sampling time points are indicated above the lanes. A previously produced C1-produced GcHS protein was used as a positive control (300ng or 1µg, respectively).

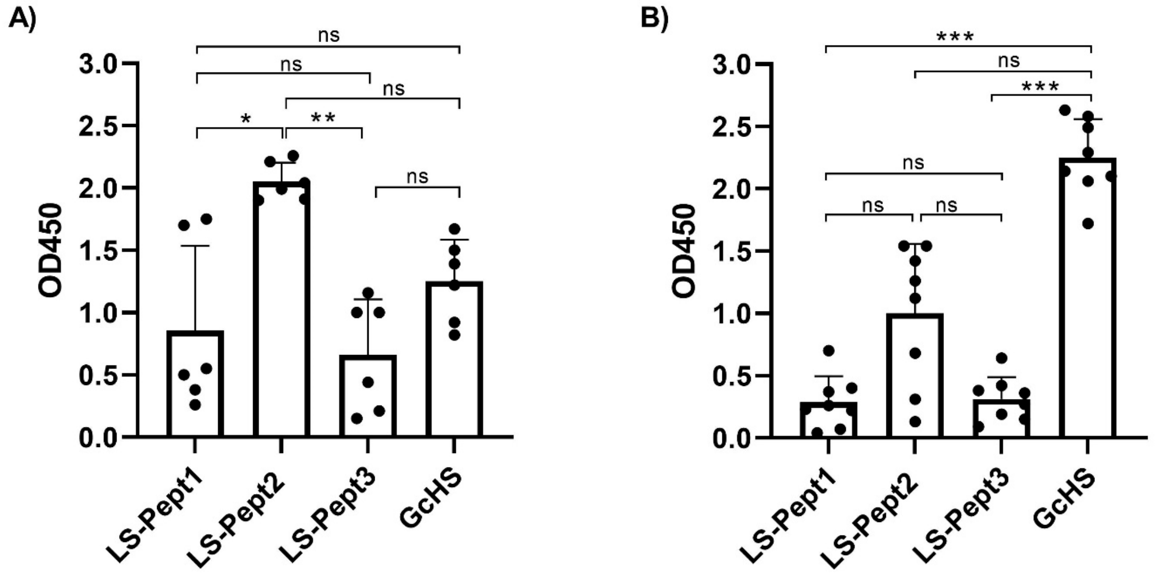

Figure S2. ELISA reactivity of peptide epitopes identified by Pepscan analysis. Three potentially immunogenic epitopes detected by Pepscan analysis were genetically fused to the LS scaffold. The resulting particles were coated on ELISA plates and their reactivity with sera from sheep (A) and cattle (B) that had recoverd from SBV infection was tested by ELISA. The recombinant SBV GcHS was included as a positive control for ELISA-reactivity. Statistical analysis was performed using the Kruskal-Wallis test followed by Dunn´s test for comparisons between individual groups. P values < 0.05 were considered significant. (* P < 0.05; ** P < 0.01; *** P < 0.001).

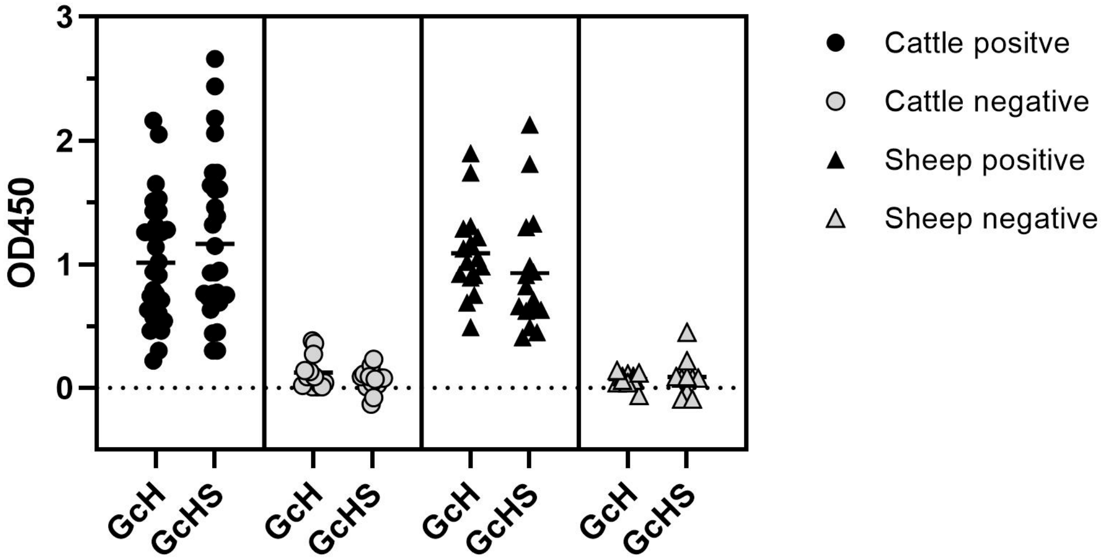

Figure S3. Comparative ELISA reactivity of the *S2*-produced SBV-GcH (GcH) and the C1-produced GcHS (GcHS) domains with SBV-antibody positive or negative serum samples.

Table S1. Summary of immunogenicity trials with an overview of vaccines, doses and animals

Trial #	Immunogen	Abbrevation	Application groups	Dose (antigen)	Challenge dose	Animals/group
1	Monomeric Gc head	GcH	2x + adjuvant	20 µg (s.c.)[1]	10^4 $TCID_{50}$ per animal (s.c)	9 IFNAR-/- mice
	Gc head conjugated to LS (unsaturated)	LS-GcH	1x + adjuvant<br>2x w/o adjuvant			
	Peptide #2 fused into LS	LS-Pept2	2x + adjuvant	20 µg (s.c.)		
2	Monomeric Gc head	GcH	2x + adjuvant	20 µg (s.c.)	$10^{6.4}$ $TCID_{50}$ per animal (s.c)	9 IFNAR-/- mice
	Gc head conjugated to LS; saturated	Sat LS-GcH	1x + adjuvant			
	Gc head conjugated to LS; unsaturated	Unsat LS-GcH				
3	Monomeric Gc head-stalk	GcHS	2x + adjuvant	20 µg (s.c.)	10^4 TCID50 per animal (s.c.)	9 IFNAR-/- mice
			1x + adjuvant			
	Gc head-stalk conjugated to LS	LS-GcHS				
4	Gc head conjugated to LS	LS-GcH	2x + adjuvant	50 µg (s.c.)	1ml infectious serum per animal (s.c.)	4 calves
	Gc head-stalk conjugated to LS	LS-GcHS				
	Peptide fused in LS	LS-Pept2				

[1] s.c. subcutaneously

Table S2. Statistical analysis of survival curves shown in Figure 4 D) and F)

Group	Versus group	Difference between groups [1]
GcH 2x + A	mock	ns
	LS-GcH 2x + A	ns
	LS-Pept2	*
	GcH 1x + A	ns
	LS-GcH 1x + A	ns
	GcH 2x w/o A	ns
	LS-GcH 2x w/o A	ns
LS-GcH 2x + A	mock	*
	GcH 2x + A	ns
	LS-Pept2	ns
	GcH 1x + A	**
	LS-GcH 1x + A	ns
	GcH 2x w/o A	*
	LS-GcH 2x w/o A	*
LS-Pept2	mock	***
	GcH 2x + A	*
	LS-GcH 2x + A	ns
	GcH 1x + A	****
	LS-GcH 1x + A	ns
	GcH 2x w/o A	***
	LS-GcH 2x w/o A	**
GcH 1x + A	mock	ns
	GcH 2x + A	ns
	LS-GcH 2x + A	**
	LS-Pept2	****
	LS-GcH 1x + A	**
	GcH 2x w/o A	ns
	LS-GcH 2x w/o A	ns
LS-GcH 1x + A	mock	*
	GcH 2x + A	ns
	LS-GcH 2x + A	ns
	LS-Pept2	****
	GcH 1x + A	**
	GcH 2x w/o A	ns
	LS-GcH 2x w/o A	*
GcH 2x w/o A	mock	ns
	GcH 2x + A	ns
	LS-GcH 2x + A	*
	LS-Pept2	***
	GcH 1x + A	ns
	LS-GcH 1x + A	*
	LS-GcH 2x w/o A	ns
LS-GcH 2x w/o A	mock	ns
	GcH 2x + A	ns

LS-GcH 2x + A	ns
LS-Pept2	**
GcH 1x + A	ns
LS-GcH 1x + A	*
GcH 2x w/o A	ns

[1] Mantel-Cox test (ns 0,1234; *0.0332; **0.0021; ***0.0002; ****<0.0001).

Table S3. Statistical analysis of survival curves shown in Figure 5 D) and F)

Group	versus group	Difference between groups [1]
GcH 2x + A	mock	ns
	Sat. LS-GcH 2x + A	*
	unsat. LS-GcH 2x + A	*
	GcH 1x + A	ns
	Sat. LS-GcH 1x + A	ns
	unsat. LS-GcH 1x + A	*
Sat. LS-GcH 2x + A	mock	**
	GcH 2x + A	*
	unsat. LS-GcH 2x + A	ns
	GcH 1x + A	**
	Sat. LS-GcH 1x + A	ns
	unsat. LS-GcH 1x + A	ns
unsat. LS-GcH 2x + A	mock	**
	GcH 2x + A	*
	Sat. LS-GcH 2x + A	ns
	GcH 1x + A	**
	Sat. LS-GcH 1x + A	ns
	unsat. LS-GcH 1x + A	ns
GcH 1x + A	mock	ns
	GcH 2x + A	ns
	Sat. LS-GcH 2x + A	**
	unsat. LS-GcH 2x + A	**
	Sat. LS-GcH 1x + A	ns
	unsat. LS-GcH 1x + A	**
Sat. LS-GcH 1x + A	mock	ns
	GcH 2x + A	ns
	Sat. LS-GcH 2x + A	ns
	unsat. LS-GcH 2x + A	ns
	GcH 1x + A	ns
	unsat. LS-GcH 1x + A	ns
unsat. LS-GcH 1x + A	mock	**
	GcH 2x + A	*
	Sat. LS-GcH 2x + A	ns
	unsat. LS-GcH 2x + A	ns
	GcH 1x + A	**
	Sat. LS-GcH 1x + A	ns

[1] Mantel-Cox test (ns 0,1234; *0.0332; **0.0021; ***0.0002; ****<0.0001).

Table S4. Statistical analysis of survival curves shown in Figure 6 D).

Group	versus group	Difference between groups [1]
GcHS 2x + A	mock	***
	GcHS 1x + A	*
	LS-GcHS 2x + A	ns
	LS-GcHS 1x + A	ns
GcHS 1x + A	mock	ns
	GcHS 2x + A	*
	LS-GcHS 2x + A	**
	LS-GcHS 1x + A	**
LS-GcHS 2x + A	mock	****
	GcHS 2x + A	ns
	GcHS 1x + A	**
	LS-GcHS 1x + A	ns
LS-GcHS 1x + A	mock	****
	GcHS 2x + A	ns
	GcHS 1x + A	**
	LS-GcHS 2x + A	ns

[1] Mantel-Cox test (ns 0,1234; *0.0332; **0.0021; ***0.0002; ****<0.0001).
